# Assessing the performance of regular surgical nose masks as a sampling method for SARS-CoV-2 detection in a cross-sectional study

**DOI:** 10.1371/journal.pone.0293001

**Published:** 2023-10-17

**Authors:** Millicent Opoku, Elizabeth Obeng-Aboagye, Georgina Yaa Kwartemaa Boamah, Dina Adu-Asamoah, Rahmat bint Yusif Ismail, Margaret Sena Akpo, Elizabeth Etornam Dogbatse, Joseph Abraham, John Kofi Odoom, Irene Owusu Donkor, Jewelna Akorli

**Affiliations:** 1 Department of Parasitology, Noguchi Memorial Institute for Medical Research, University of Ghana, Legon, Accra, Ghana; 2 Department of Virology, Noguchi Memorial Institute for Medical Research, University of Ghana, Legon, Accra, Ghana; 3 Department of Epidemiology, Noguchi Memorial Institute for Medical Research, University of Ghana, Legon, Accra, Ghana; University of Ghana Medical School, GHANA

## Abstract

Nose masks are widely worn for protection against respiratory pathogens, including SARS-CoV-2. They have been reported as possible substrates for viral sampling and testing for COVID-19 but, evaluations have so far been purposive; involving individuals known to have the infection and using improved materials on the nose masks to trap the virus. We investigated the feasibility of using the regular 3-ply surgical masks and, voluntary coughing as a mode of particle expulsion for detecting SARS-CoV-2 infections in a cross-sectional study at Ghana’s first COVID-19 testing reference laboratory, the Noguchi Memorial Institute for Medical Research, University of Ghana. Paired samples of naso-oropharyngeal swabs and nose masks already worn by 103 consenting adult participants (retro masks) were collected. Participants were also required to produce three strong coughs into a newly supplied sterile surgical nose mask. Pre-wetted swabs in Viral Transport Media (VTM) were used in swabbing the inner lining of each nose mask. The swabs used were then stored in VTM to maintain the integrity of the samples. PCR results of SARS-CoV-2 detection from the nose masks were compared to those from naso-oropharyngeal swabs (‘gold-standard’). Out of the 103 participants tested with all three methods, 66 individuals sampled with naso-oropharyngeal swabs were detected as positive, and the retro and new masks matched 9 and 4, respectively. Only 3 individuals were positive across all three sampling methods accessed. The retro nose masks performed better in matching the gold-standard results than the new mask + coughing method, with 90% vs 80% sensitivity, positive predictive value of 13.6% vs 6%, and a weak but significant linear relationship (adj. R^2^ = 0.1; *P* = 0.0004). Importantly, we also show that the nose masks would work for sampling whether individuals are symptomatic or asymptomatic since gold-standard PCR cycling threshold (Ct) values for positive individuals did not differ between the two groups (*P*< 0.05). We recommend including features such as talking during participant engagement, use of a spontaneous cough inducer and increased coughing bouts > 3, to improve the performance of sterile nose masks for SARS-CoV-2 detection.

## Introduction

The major route for SARS-CoV-2 transmission is through air-borne respiratory droplets. The virus enters the human body primarily via the nasal cavity and resides in epithelial cells to establish infection. Viral replication occurs at this site and the virus remains detectable in the upper respiratory tract for several weeks. The gold-standard sampling method for detecting SARS-CoV-2 infection is swabbing the naso-oropharyngeal cavity and performing quantitative RT-PCR on extracted viral material. Cycle threshold (Ct) values are often used as a proxy for viral load [1] and are postulated to be inversely related to a person’s infectiousness and transmissibility [2]. At Ct values >32, hospitalized patients are considered non-infectious and are discharged [3]. However, concerns have been raised about this criterion since viral particles isolated from individuals with Ct values >30 are culturable and suggest they could be infectious [2].

Major guidelines established to help reduce transmission of SARS-CoV-2 were focused on minimizing the proximity within which aerosols could be transferred from environment to people, wearing of nose/face masks and observing social/physical distancing. Nose masks are still recommended in certain situations as new cases of SAR-CoV-2 infections are still being reported [4]. Nose masks serve as barriers that trap viral particles from infected persons especially when such people talk, cough or sneeze thus preventing the transfer of the infectious agent to others. It also reduces the risk of infection in uninfected individuals when worn properly to cover both mouth and nose. The viral load captured on a used nose mask may therefore offer a better measure of the potential for one to transmit the virus rather than estimates from naso-oropharyngeal swabs [5], as it would depict how much virus is being released. Indeed, nose masks have been used as fomites to sample other respiratory pathogens [6, 7] and recently for SARS-CoV-2 [5, 8, 9].

Although reports are suggestive of the potential use of nose masks for COVID-19 diagnosis and other epidemiological interpretations such as transmissibility [5], these have mainly been purposive, based on hospitalized symptomatic patients. Evaluation of the efficiency of nose masks as sampling tools is required, especially in its ability to detect asymptomatic infections. We tested the reported use of the more commonly worn surgical nose masks as an alternative tool for sampling at a COVID-19 testing centre. We collected worn nose masks from participants and provided new ones to test voluntary coughing bouts as a viral particle expelling method for clinical sampling of SARS-CoV-2 using masks.

## Methods

### Ethical statement

This study was conducted at the COVID-19 testing centre of Noguchi Memorial Institute for Medical Research, University of Ghana in June-July 2022. Ethical approval (NMIMR-IRB CPN# 025/21-22) was obtained from the Noguchi Institutional Ethical Review Board (Federal Wide Assurance #: 00001824).

Written informed consent was obtained from eligible persons visiting the testing centre. The objectives of the study were explained to individuals in simple lay language. Potential participants were allowed time to read the consent form and ask questions concerning the study and sign the form if they agree to participate. Only consenting adults (>17 years) were included as participants. Since all adults are eligible to test for COVID-19 and, the method being tested was non-invasive, no exclusion criteria were considered.

The authors involved in conducting the COVID-19 tests had access to information that could identify participants during the study, since test results had to be reported back to clients. However, each participant was assigned a unique ID to ensure anonymity after the provision of standard COVID-19 test results, and for subsequent analyses for this study.

### Sample size

We arrived at a sample size of 95, calculated based on the reported number of clients that visited the testing centre daily (~100) and the positivity rate (5.7%) at the time of conducting the study. Due to the sensitivity of polymerase chain reaction (PCR), the acceptable margin of error was set to 1% and confidence level at 95%.

Sample size was calculated using the formula:

$$n=Deff\times strata\times \frac{Z_{1-\frac{\alpha }{2}}^{2}P(1-P)}{e^{2}(1-NR)}$$

Where Deff is the designed effect, strata is the number of study areas, $Z_{1-\frac{\alpha }{2}}^{2}$ is the square of the standard normal variate, NR is the non-response rate, P is the prevalence of SARS-CoV-2.

### Sample collection

A total of 103 persons who were clients at the NMIMR testing centre between June-July 2021 consented to participate in the study. Once informed consent was obtained, a simple questionnaire was administered to collect demographic data and other information relevant to the study; including the reasons a test was requested. Throughout the period of engagement with the participant before naso-oropharngygeal swab sample was taken, participants had their masks on. It was a requirement to wear nose masks when entering the testing centre. These nose masks designated as ‘retro’ nose mask were obtained from the participants before naso-oropharyngeal (NOP) swabs were taken. Participants were then provided with a new sterile nose mask. Once worn, nose and mouth areas of the masks were marked on the outside. Each participant was requested to produce 3 strong coughs while wearing the mask, after which the masks were also collected. NOP swabs were then taken normally as standard protocol for COVID-19 sampling and, fresh nose masks were given to the participants to wear before leaving the testing centre. The inner lining of the marked area of each nose mask retrieved was swabbed with a swab pre-wetted in viral transport medium (VTM) (Biobase Biotech, Jinan, China). The ‘new’ and the retro masks (though not marked), were swabbed similarly. Each of the two nose mask swabs was returned to a separate tube of VTM (Biobase Biotech, Jinan, China) labelled with participant ID. Nose masks were stored in well-labelled Ziplock bags at -20°C. All swabs were immediately processed for SARS-CoV-2 detection.

### Sample processing

The QIAamp Viral RNA Mini Kit (Qiagen Corporation, Germany) was used to extract viral RNA from both naso-oropharyngeal and nose mask swabs according to the manufacturer’s instructions. A no-template control was included in the extraction process to check for contamination. Quantitative RT-PCR was carried out on the eluted RNA using the Veri-Q PCR 316 COVID-19 Detection Kit targeting the ORF3a and N-gene (MiCo Biomed Corporation, South Korea) and, using the commercially provided positive control, the in-house no-template extraction control and a no-template qPCR control to validate the assays.

### Data analyses

PCR results of SARS-CoV-2 detection from nose masks were compared to those from the gold standard sampling method (NOP swabs). A two-tailed Fisher’s Exact Test for count data was performed to test for association between categorical variables. Median Ct values are reported with lower and upper limits. A linear regression model was fitted to pairwise Ct data to determine the relationship between the tests. Sensitivity, specificity, positive and negative predictive values for the nose mask tests were determined using package *epiR* [10] in R.

## Results

**Fig 1:**
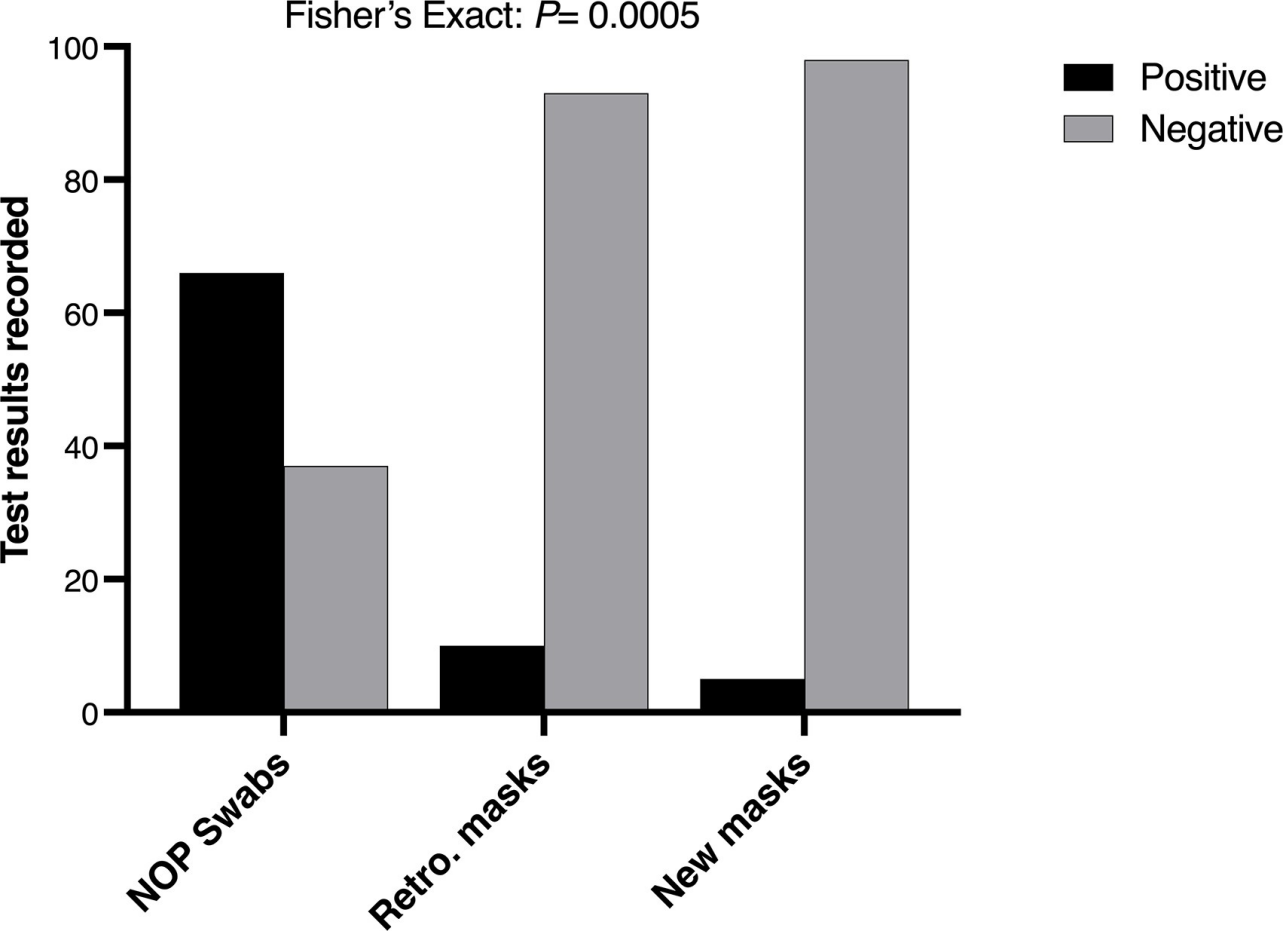
Results of SARS-CoV-2 detection from 103 naso-oropharyngeal swabs and matched nose masks. *P*-value for the Fisher’s exact test was performed based on Monte Carlo simulation.

**Fig 2:**
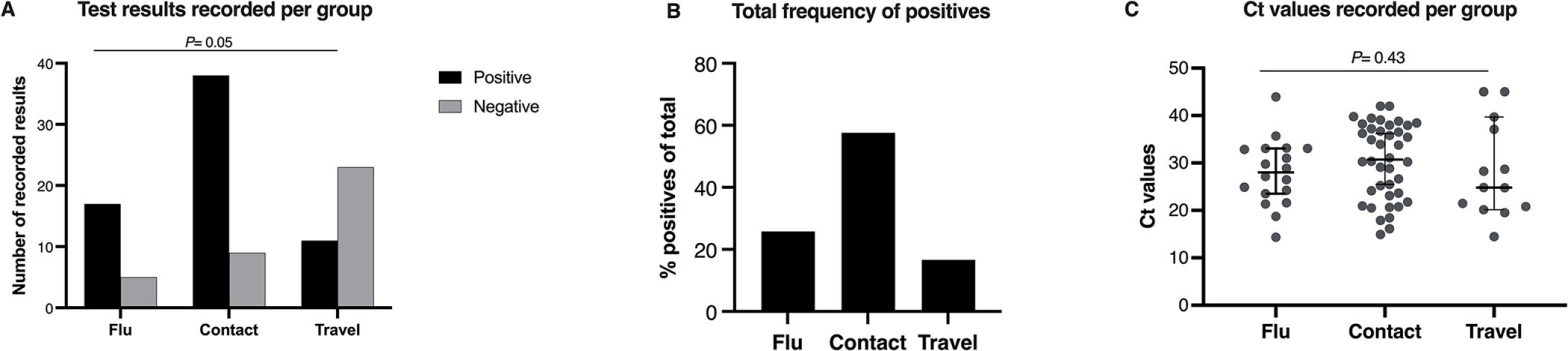
Results of SARS-CoV-2 infection in study participants using the gold-standard naso-oropharyngeal swab method. Plots are grouped by participants’ reasons for taking the test. The bar plots of positives and negatives (A) included all results reported following RT-PCR. ie. numeric Ct value and ‘undetermined’ (classified as negative). The proportion of positives detected from the reasons for getting the test (B) and Fisher’s exact test was used to test if infection was dependent on whether one shows symptoms or not (*P*>0.05). Each dot in ‘C’ represents individual Ct values. Only numeric values from RT-PCR were used (excluded ‘undetermined’). Test of significance was determined with Kruskal-Wallis.

A total of 103 complete data for naso-oropharyngeal swabs and two nose masks per participant were obtained (S1 Table). Sixty-six (66) NOP swabs showed positive for SARS-CoV-2, representing a positivity rate of 64% while each of the nose masks recorded less than 10% positivity (Fig 1). There was significant association between infection results and the sampling method (Fisher’s exact: *P*<0.0005), suggesting that detecting positivity depended on what sampling method was used. More than 50% of the positives detected using the gold-standard method were individuals who reported having contact with an infected person, but exhibited no clinical symptoms (Fig 2A and 2B). This was however of marginal significance (Fisher’s exact: *P* = 0.05). Interestingly, there was no difference in the median Ct values of positive individuals with flu symptoms and those who were asymptomatic (contact with an infected person and for travel) (*P* = 0.43) (Fig 2C), suggesting that both groups have similar viral loads and transmissibility.

**Fig 3:**
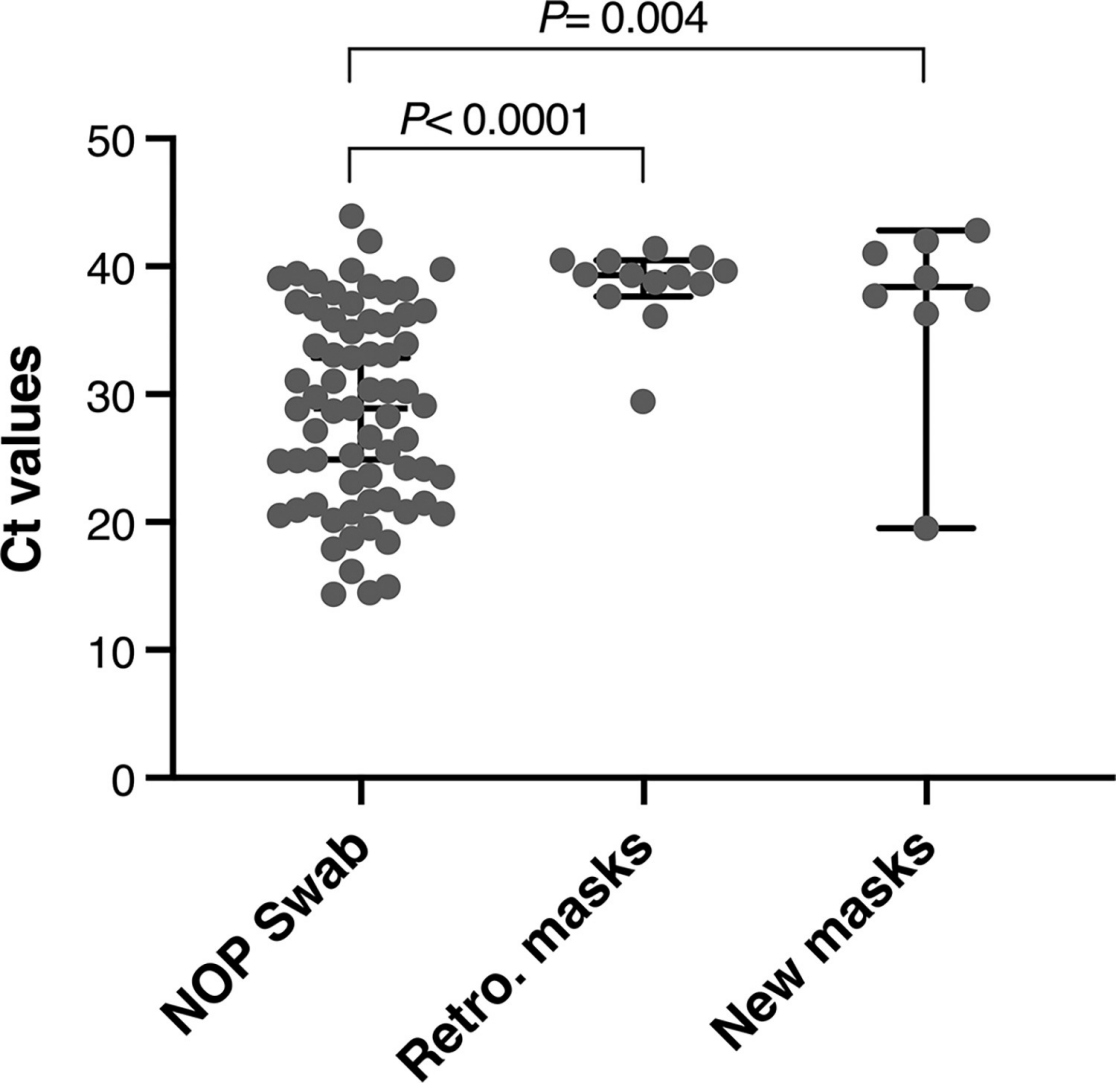
Cycle threshold (Ct) values RT-PCR detection of SARS-CoV-2 from naso-oropharyngeal (NOP) swabs and matched nose masks. Each point is a Ct value result provided following the RT-PCR reaction. Results that were given as ‘undetermined’ are not plotted. Horizontal line represents the median Ct and error bars are 95% confidence intervals. *P*-values are two-tailed from Mann Whitney tests.

The Ct values observed from NOP swabs (median = 28.8; 21.6–35.8) were lower than from the nose masks (retro = 39.5, new = 38.4) (*P*< 0.05) (Fig 3). 84% (87/103) and 92% (95/103) of retro and new masks, respectively, recorded ‘undetermined’ Ct results compared to 34% (35/103) for NOP swabs (S1 Table).

**Fig 4:**
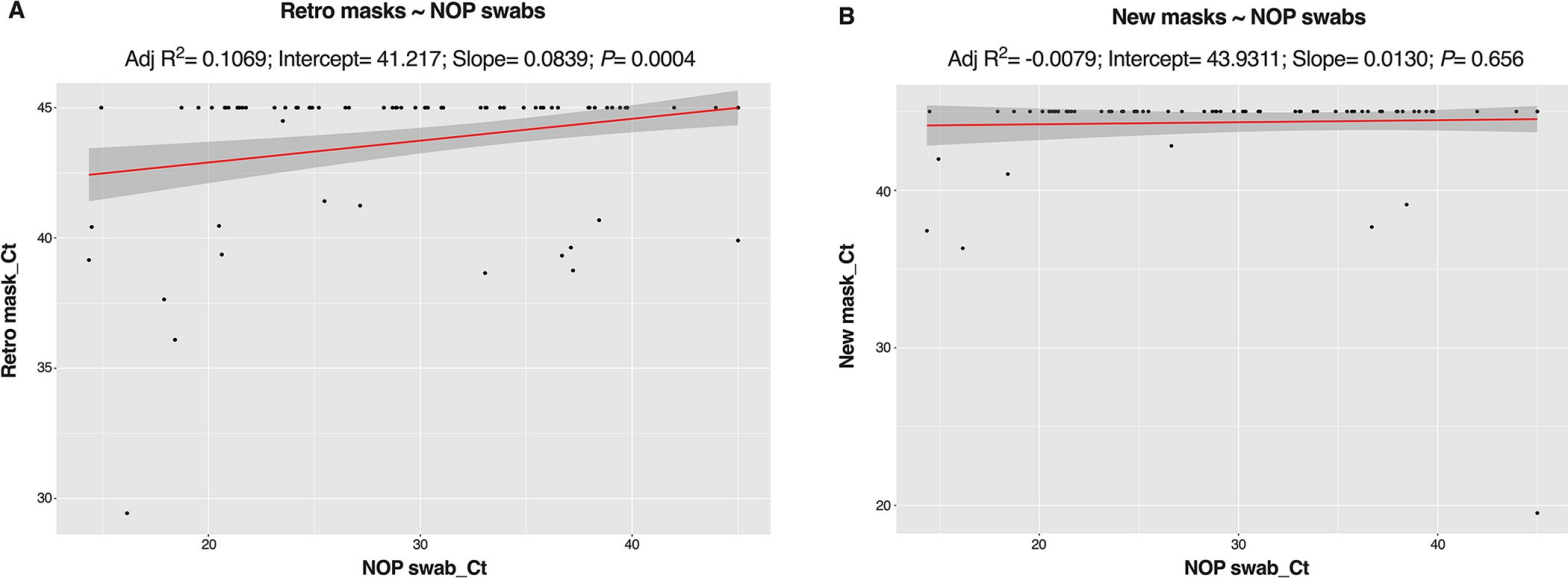
Linear regression model plots for nose mask Ct values as a response to matched naso-oropharyngeal (NOP) swab Ct. *P*-values show the significance of the coefficient estimate (slope).

We fitted a simple linear regression model to determine possible relationships between the Ct values obtained for the tests. For this, we assigned all ‘undetermined’ a value of 45; the number of cycles run for the RT-PCR. The retro masks better matched values obtained from the NOP swabs and showed a stronger relationship (adjusted R-squared = 0.1, F-statistic = 13.2) compared to that between NOP and new masks (adj R-squared = -0.008, F-statistic = 0.2) (Fig 4, S2 Table). The effect Ct value that can be observed using the retro mask is 0.08 times higher (coefficient estimate = 0.08, *P* = 0.0004) than values from the gold standard (NOP). Although the coefficient estimate from the new masks was lower (coefficient estimate = 0.01), this was not significant (*P* = 0.66) (S2 Table).

**Fig 5:**
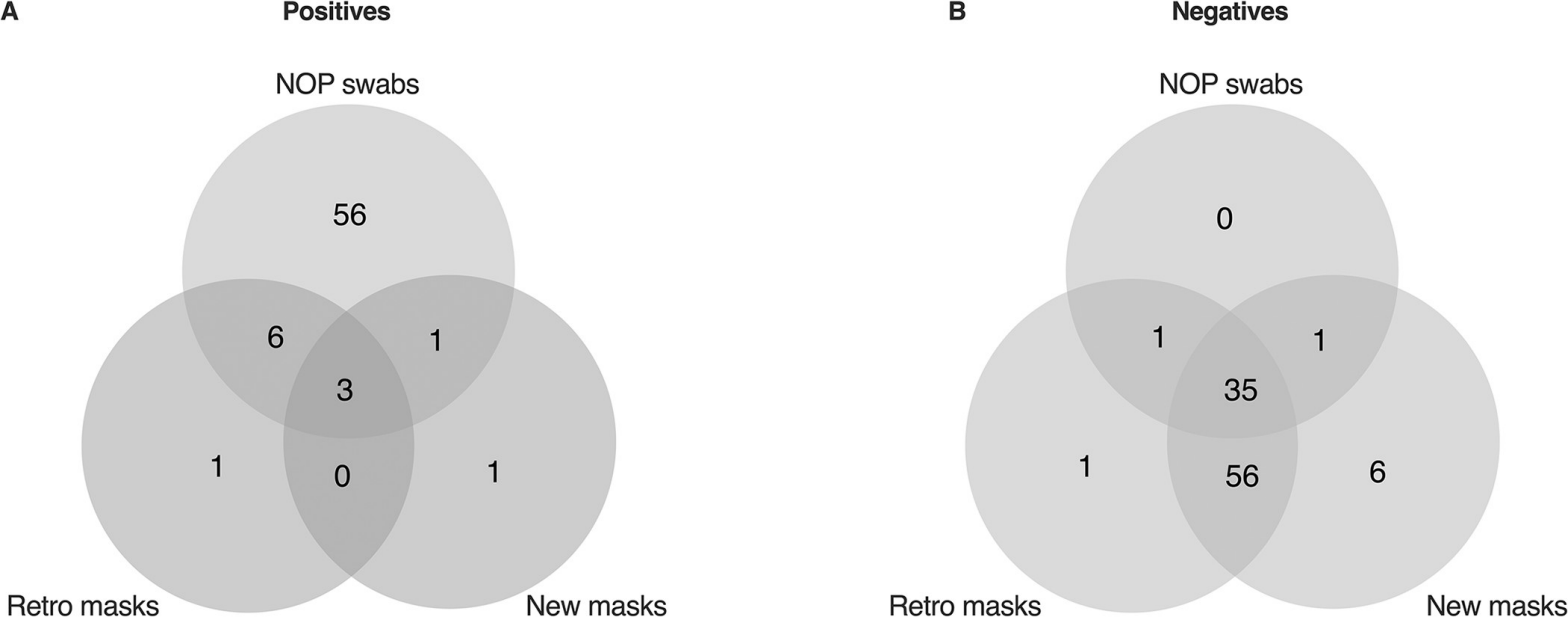
Venn diagram for matched SARS-CoV-2 results from the three test methods.

The comparisons were next evaluated based on their qualitative test results. NOP swabs and nose masks shared a relatively small number of positive outcomes compared to negatives (Fig 5). Only 11% (7/66) of NOP swab positives were detected by either nose masks and 5% (3/66) by matched retro and new masks (Fig 5A). Sensitivity (the ability to correctly identify persons with infection) was estimated to be 90% (95% CI: 55% - 100%) and 80% (95% CI: 28% - 99%) for retro and new nose masks, respectively (Table 1). Specificity (the ability to correctly identify persons without infection) was similar for both masks. Diagnoses using the retro and new masks was 44% and 39% accurate, respectively. Based on the results, twice the number of persons tested with retro masks will need to be tested with the new mask method to capture the same number of positive individuals in a study population (S3 Table: nndx: retro = 3.48, new = 5.98).

**Table 1:** Point estimates with 95% confidence intervals (CI) of parameters comparing the performance of nose masks against gold-standard naso-oropharyngeal swabs for SARS-CoV-2 detection. Cross- tab tables of result count data were analysed with package *epiR* [10]. ‘Test’ refers to the NOP swabs and ‘outcome’ refers to masks.

Parameter	Retro masks	New masks
Sensitivity	0.90 (0.55, 1.00)	0.80 (0.28, 0.99)
Specificity	0.39 (0.29, 0.49)	0.37 (0.27, 0.47)
Positive predictive value	0.14 (0.06, 0.24)	0.06 (0.02, 0.15)
Negative predictive value	0.97 (0.86, 1.00)	0.97 (0.86, 1.00)
Positive likelihood ratio	1.47 (1.13, 1.91)	1.26 (0.80, 2.01)
Negative likelihood ratio	0.26 (0.04, 1.69)	0.54 (0.09, 3.20)
True outcome negative subjects that test positive	0.61 (0.51, 0.71)	0.63 (0.53, 0.73)
True outcome positive subjects that test negative	0.10 (0.00, 0.45)	0.20 (0.01, 0.72)
Test positive subjects that are outcome negative	0.86 (0.76, 0.94)	0.94 (0.85, 0.98)
Test negative subjects that are outcome positive	0.03 (0.00, 0.14)	0.03 (0.00, 0.14)
Correctly classified proportion	0.44 (0.34, 0.54)	0.39 (0.29, 0.49)

## Discussion

Nose masks remain appropriate for preventing transmission of respiratory droplets, and could be developed as a non-invasive sampling tool for testing respiratory pathogens including SARS-CoV-2. We tested voluntary coughing into regular surgical nose masks as a possible virus sampling method. This method was expected to produce instant viral particle expulsion from the oropharynx [11]. It was assumed to be more practical for use in a typical testing set-up than asking individuals to wear the nose mask for a specific period while performing activities such as talking, singing, or wait to sneeze or cough involuntarily [5, 6, 8]. We evaluated both scenarios by including masks that were already worn by participants ie. retro nose masks, but information on how long the masks had been worn or what potential virus expelling activities had been performed while wearing the masks was not captured. Collecting such data would have heavily relied on participant memory recall of involuntary activities and led to false data.

The higher diagnostic accuracy, sensitivity, and likelihood ratio of a positive test estimated for the retro masks support previous results that there is an increased chance of trapping viral particles when more than one virus expelling activity is performed while wearing the mask [5, 8, 9]. The retro masks generally performed better than the new masks which only captured coughs and some talking, when participants felt the need to speak while the new masks had been worn. The estimated number of individual masks needed to diagnose also purports that collecting retro masks in a cross-sectional study, for example, may be better at detecting positives in an epidemiological survey. However, caution need to be taken if retro masks are to be used in such a study as length of time each participant has worn the mask, whether the mask has not been shared, and the activities that have been performed into the masks are likely confounding factors. To avoid this, we propose including talking into the nose masks, which could be done during questionnaire administration after consenting, to our coughing method (3 times) to improve the performance of using new, sterile masks for sampling.

Different forms of masks became available during the peak of the pandemic when mask-wearing was established among the protective guidelines. KN95 masks were reported as the most protective as the material used can trap viral particles more efficiently [12]. Although the regular surgical masks are known to be less effective against tiny viral droplets [13] they are the most used as they are less expensive and more readily available. In this study, we used these surgical nose masks to assess the tool in its most commonly available form to the study population. It is important to note that only 5 study participants walked into the testing centre wearing a KN95 mask, thus 95% of the retro masks were surgical masks similar to the new masks provided. While materials including gelatin have proven effective in trapping viral particles [5], we have shown that the ordinary 3-ply surgical nose mask is also effective, providing sensitivity between 80–90%. This can be improved with a more effective way of isolating the virus from the inner ply of the masks rather than swabbing as was done in this current study. The inner ply could be cut out after defining the area in proximity to the mouth and nose, submerging into an appropriate medium and performing viral extraction. The amount of medium would need optimization to ensure the virus is not diluted out of detection. Furthermore, the efficiency of using nose masks for testing respiratory viruses may be dependent on several factors including the capacity of material from which the nose mask was made to trap viral particles, the method used to expel viruses from the host, the viral retrieval and isolation method. These approaches need to be carefully considered, defined, and standardized before nose masks can be accepted as a tool for sampling [14].

## Conclusion

Our results have reiterated the possible use of simple surgical nose masks for COVID-19 testing. We recommend standardizing talking and coughing as the voluntary expiratory activities to be used. Compared to other studies that assessed nose masks while purposefully selecting confirmed infected persons and matching with uninfected persons from hospital setting, our study sampled persons without *a priori* knowledge of their infection status. This makes our study more random and cross-sectional. We had a choice of either being stationed or going out to the public to sample from the streets, household etc. However, we decided to test our hypothesis using the simplest approach, which was sampling people visiting the testing centre. Further studies could do broader population sampling.

## Supporting information

S1 Checklist STROBE statement—checklist of items that should be included in reports of observational studies. (DOCX)

S1 Table Sample information from 103 participants. (XLSX)

S2 Table Results for linear regression model for Ct values between test methods. (DOCX)

S3 Table Detailed statistical estimates for sensitivity, specificity and predictive prevalence analyses of nose masks compared to naso-oropharyngeal swabs. (DOCX)
