# Supplementary material for: Assessing the performance of regular surgical nose masks as a sampling method for SARS-CoV-2 detection in a cross-sectional study
Source: PLoS One. 2023 Oct 17;18(10):e0293001. doi: 10.1371/journal.pone.0293001 (PMC10581487; doi:10.1371/journal.pone.0293001)
Supplement: S1 Checklist — (DOCX) [file pone.0293001.s001.docx]

STROBE Statement—checklist of items that should be included in reports of observational studies

|  | Item No. | Recommendation | Page  No. | Relevant text from manuscript |
| --- | --- | --- | --- | --- |
| **Title and abstract** | 1 | (*a*) Indicate the study’s design with a commonly used term in the title or the abstract | 1 | Assessing the performance of regular surgical nose masks as a sampling method for SARS-COV-2 detection in a **cross-sectional study** |
|  |  | (*b*) Provide in the abstract an informative and balanced summary of what was done and what was found | 2 | Paired samples of naso-oropharyngeal swabs and nose masks already worn by 103 consenting adult participants (retro masks) were collected. Participants were also required to produce three strong coughs into a newly supplied sterile surgical nose mask. PCR results of SARS-CoV-2 detection from the nose masks were compared to those from naso-oropharyngeal swabs (‘gold-standard’). Out of the 103 participants tested with all three methods, 66 individuals sampled with naso-oropharyngeal swabs were detected as positive, and the retro and new masks matched 9 and 4, respectively. Only 3 individuals were positive across all three sampling methods accessed. The retro nose masks performed better in matching the gold-standard results than the new mask + coughing method, with 90% vs 80% sensitivity, positive predictive value of 13.6% vs 6%, and a weak but significant linear relationship (adj. R^2^= 0.1; *P*= 0.0004). Importantly, we also show that the nose masks would work for sampling whether individuals are symptomatic or asymptomatic since gold-standard PCR cycling threshold (Ct) values for positive individuals did not differ between the two groups (*P*< 0.05). |
| Introduction | | | |  |
| Background/rationale | 2 | Explain the scientific background and rationale for the investigation being reported | 3-4 | The major route for SARS-CoV-2 transmission is through air-borne respiratory droplets. The virus enters the human body primarily via the nasal cavity and resides in epithelial cells to establish infection. Viral replication occurs at this site and the virus remains detectable in the upper respiratory tract for several weeks. The gold-standard sampling method for detecting SARS-CoV-2 infection is swabbing the naso-oropharyngeal cavity and performing quantitative RT-PCR on extracted viral material. Ct values are often used as a proxy for viral load (1) and are postulated to be inversely related to a person’s infectiousness and transmissibility (2). At Ct values >32, hospitalized patients are considered non-infectious and are discharged (3). However, concerns have been raised about this criterion since viral particles isolated from individuals with Ct values >30 are culturable and suggest they could be infectious (2).  Major guidelines established to help reduce transmission of SARS-CoV-2 were focused on minimizing the proximity within which aerosols could be transferred from environment to people, wearing of nose/face masks and observing social/physical distancing. Nose masks are still recommended in certain situations as new cases of COVID-19 infections are still being reported (4). Nose masks serve as barriers that trap viral particles from infected persons especially when such people talk, cough or sneeze thus preventing the transfer of the infectious agent to others. It also reduces the risk of infection in uninfected individuals when worn properly to cover both mouth and nose. The viral load captured on a used nose mask may therefore offer a better measure of the potential for one to transmit the virus rather than estimates from naso-oropharyngeal swabs (5), as it would depict how much virus is being released. Indeed, nose masks have been used as fomites to sample other respiratory pathogens (6,7) and recently for SARS-CoV-2 (5,8,9).  Although reports are suggestive of the potential use of nose masks for COVID-19 diagnosis and other epidemiological interpretations such as transmissibility (5), these have mainly been purposive, based on hospitalized symptomatic patients. Evaluation of the efficiency of nose masks as sampling tools is required, especially in its ability to detect asymptomatic infections. We tested the reported use of the more commonly worn surgical nose masks as an alternative tool for sampling at a COVID-19 testing centre. We collected worn nose masks from participants and provided new ones to test voluntary coughing bouts as a viral particle expelling method for clinical sampling of SARS-CoV-2 using masks. |
| Objectives | 3 | State specific objectives, including any prespecified hypotheses | 4 | Although reports are suggestive of the potential use of nose masks for COVID-19 diagnosis and other epidemiological interpretations such as transmissibility (5), these have mainly been purposive, based on hospitalized symptomatic patients. Evaluation of the efficiency of nose masks as sampling tools is required, especially in its ability to detect asymptomatic infections. We tested the reported use of the more commonly worn surgical nose masks as an alternative tool for sampling at a COVID-19 testing centre. We collected worn nose masks from participants and provided new ones to test voluntary coughing bouts as a viral particle expelling method for clinical sampling of SARS-CoV-2 using masks. |
| Methods | | | |  |
| Study design | 4 | Present key elements of study design early in the paper | 4 | We tested the reported use of the more commonly worn surgical nose masks as an alternative tool for sampling at a COVID-19 testing centre. We collected worn nose masks from participants and provide new ones to test voluntary coughing bouts as a viral particle expelling method for clinical sampling of SARS-CoV-2 using masks. |
| Setting | 5 | Describe the setting, locations, and relevant dates, including periods of recruitment, exposure, follow-up, and data collection | 4 | This study was conducted at the COVID-19 testing centre of Noguchi Memorial Institute for Medical Research, University of Ghana in June-July 2022. |
| Participants | 6 | (*a*) *Cohort study*—Give the eligibility criteria, and the sources and methods of selection of participants. Describe methods of follow-up  *Case-control study*—Give the eligibility criteria, and the sources and methods of case ascertainment and control selection. Give the rationale for the choice of cases and controls  *Cross-sectional study*—Give the eligibility criteria, and the sources and methods of selection of participants | 5 | A total of 103 persons who were clients at the NMIMR testing centre between June-July 2021 consented to participate in the study. Once informed consent was obtained, a simple questionnaire was administered to collect demographic data and other information relevant to the study; including the reasons a test was requested. |
|  |  | (*b*) *Cohort study*—For matched studies, give matching criteria and number of exposed and unexposed  *Case-control study*—For matched studies, give matching criteria and the number of controls per case |  |  |
| Variables | 7 | Clearly define all outcomes, exposures, predictors, potential confounders, and effect modifiers. Give diagnostic criteria, if applicable | 6 | PCR results of SARS-CoV-2 detection from nose masks were compared to those from the gold standard sampling method (NOP swabs). A two-tailed Fisher’s Exact Test for count data was performed to test for association between categorical variables. Median Ct values are reported with lower and upper limits. A linear regression model was fitted to pairwise Ct data to determine the relationship between the tests. Sensitivity, specificity, positive and negative predictive values for the nose mask tests were determined using package *epiR* (10) in R. |
| Data sources/ measurement | 8* | For each variable of interest, give sources of data and details of methods of assessment (measurement). Describe comparability of assessment methods if there is more than one group |  |  |
| Bias | 9 | Describe any efforts to address potential sources of bias |  |  |
| Study size | 10 | Explain how the study size was arrived at | 5 | We arrived at a sample size of 95, calculated sample size based on the reported number of clients that visited the testing centre daily (~100) and the positivity rate (5.7%) at the time of conducting the study. Due to the sensitivity of PCR, the acceptable margin of error was set to 1% and confidence level at 95%. |

Continued on next page

| Quantitative variables | 11 | Explain how quantitative variables were handled in the analyses. If applicable, describe which groupings were chosen and why |  |  | |
| --- | --- | --- | --- | --- | --- |
| Statistical methods | 12 | (*a*) Describe all statistical methods, including those used to control for confounding | 6-7 | | PCR results of SARS-CoV-2 detection from nose masks were compared to those from the gold standard sampling method (NOP swabs). A two-tailed Fisher’s Exact Test for count data was performed to test for association between categorical variables. Median Ct values are reported with lower and upper limits. A linear regression model was fitted to pairwise Ct data to determine the relationship between the tests. Sensitivity, specificity, positive and negative predictive values for the nose mask tests were determined using package *epiR* (10) in R. |
|  |  | (*b*) Describe any methods used to examine subgroups and interactions |  | |  |
|  |  | (*c*) Explain how missing data were addressed |  | |  |
|  |  | (*d*) *Cohort study*—If applicable, explain how loss to follow-up was addressed  *Case-control study*—If applicable, explain how matching of cases and controls was addressed  *Cross-sectional study*—If applicable, describe analytical methods taking account of sampling strategy |  | |  |
|  |  | (*e*) Describe any sensitivity analyses | 7 | | Sensitivity, specificity, positive and negative predictive values for the nose mask tests were determined using package *epiR* (10) in R. |
| Results | | | | | |
| Participants | 13* | (a) Report numbers of individuals at each stage of study—eg numbers potentially eligible, examined for eligibility, confirmed eligible, included in the study, completing follow-up, and analysed |  | |  |
|  |  | (b) Give reasons for non-participation at each stage |  | |  |
|  |  | (c) Consider use of a flow diagram |  | |  |
| Descriptive data | 14* | (a) Give characteristics of study participants (eg demographic, clinical, social) and information on exposures and potential confounders |  | |  |
|  |  | (b) Indicate number of participants with missing data for each variable of interest |  | |  |
|  |  | (c) *Cohort study*—Summarise follow-up time (eg, average and total amount) |  | |  |
| Outcome data | 15* | *Cohort study*—Report numbers of outcome events or summary measures over time | 7 | | Sixty-six (66) NOP swabs showed positive for SARS-CoV-2, representing a positivity rate of 64% while each of the nose masks recorded less than 10% positivity (Fig. 1). |
|  |  | *Case-control study—*Report numbers in each exposure category, or summary measures of exposure |  | |  |
|  |  | *Cross-sectional study—*Report numbers of outcome events or summary measures |  | |  |
| Main results | 16 | (*a*) Give unadjusted estimates and, if applicable, confounder-adjusted estimates and their precision (eg, 95% confidence interval). Make clear which confounders were adjusted for and why they were included | 7-10 | | **Results** |
|  |  | (*b*) Report category boundaries when continuous variables were categorized |  | |  |
|  |  | (*c*) If relevant, consider translating estimates of relative risk into absolute risk for a meaningful time period |  | |  |

Continued on next page

| Other analyses | 17 | Report other analyses done—eg analyses of subgroups and interactions, and sensitivity analyses |  |  |
| --- | --- | --- | --- | --- |
| Discussion | | | | |
| Key results | 18 | Summarise key results with reference to study objectives | 11-12 | Nose masks remain appropriate for preventing transmission of respiratory droplets, and could be developed as a non-invasive sampling tool for testing respiratory pathogens including SARS-CoV-2. We tested voluntary coughing into regular surgical nose masks as a possible virus sampling method. This method was expected to produce instant viral particle expulsion from the oropharynx (11). It was assumed to be more practical for use in a typical testing set-up than asking individuals to wear the nose mask for a specific period while performing activities such as talking, singing, or wait to sneeze or cough involuntarily (5,6,8). We evaluated both scenarios by including masks that were already worn by participants ie. retro nose masks, but information on how long the masks had been worn or what potential virus expelling activities had been performed while wearing the masks was not captured. Collecting such data would have heavily relied on participant memory recall of involuntary activities and led to false data.  The higher diagnostic accuracy, sensitivity, and likelihood ratio of a positive test estimated for the retro masks support previous results that there is an increased chance of trapping viral particles when more than one virus expelling activity is performed while wearing the mask(5,8,9). The retro masks generally performed better than the new masks which only captured coughs and some talking, when participants felt the need to speak while the new masks had been worn. The estimated number of individual masks needed to diagnose also purports that collecting retro masks in a cross-sectional study, for example, may be better at detecting positives in an epidemiological survey. However, caution need to be taken if retro masks are to be used in such a study as length of time each participant has worn the mask, whether the mask has not been shared, and the activities that have been performed into the masks are likely confounding factors. To avoid this, we propose including talking into the nose masks, which could be done during questionnaire administration after consenting, to our coughing method (3 times) to improve the performance of using new, sterile masks for sampling.  Different forms of masks became available during the peak of the pandemic when mask-wearing was established among the protective guidelines. KN95 masks were reported as the most protective as the material used can trap viral particles more efficiently (12). Although the regular surgical masks are known to be less effective against tiny viral droplets (13) they are the most used as they are less expensive and more readily available. In this study, we used these surgical nose masks to assess the tool in its most commonly available form to the study population. It is important to note that only 5 study participants walked into the testing centre wearing a KN95 mask, thus 95% of the retro masks were surgical masks similar to the new masks provided. While materials including gelatin have proven effective in trapping viral particles (5), we have shown that the ordinary 3-ply surgical nose mask is also effective, providing sensitivity between 80-90%. This can be improved with a more effective way of isolating the virus from the inner ply of the masks rather than swabbing as was done in this current study. The inner ply could be cut out after defining the area in proximity to the mouth and nose, submerging into an appropriate medium and performing viral extraction. The amount of medium would need optimization to ensure the virus is not diluted out of detection. Furthermore, the efficiency of using nose masks for testing respiratory viruses may be dependent on several factors including the capacity of material from which the nose mask was made to trap viral materials, the method used to expel viruses from the host, the viral retrieval and isolation method. These approaches need to be carefully considered, defined, and standardized before nose masks can be accepted as a tool for sampling (14). |
| Limitations | 19 | Discuss limitations of the study, taking into account sources of potential bias or imprecision. Discuss both direction and magnitude of any potential bias | 11  12 | We evaluated both scenarios by including masks that were already worn by participants ie. retro nose masks, but information on how long the masks had been worn or what potential virus expelling activities had been performed while wearing the masks was not captured.  While materials including gelatin have proven effective in trapping viral particles (5), we have shown that the ordinary 3-ply surgical nose mask is also effective, providing sensitivity between 80-90%. This can be improved with a more effective way of isolating the virus from the inner ply of the masks rather than swabbing as was done in this current study. The inner ply could be cut out after defining the area in proximity to the mouth and nose, submerging into an appropriate medium and performing viral extraction. |
| Interpretation | 20 | Give a cautious overall interpretation of results considering objectives, limitations, multiplicity of analyses, results from similar studies, and other relevant evidence | 12 | These approaches need to be carefully considered, defined, and standardized before nose masks can be accepted as a tool for sampling (14). |
| Generalisability | 21 | Discuss the generalisability (external validity) of the study results |  |  |
| Other information | |  | | |
| Funding | 22 | Give the source of funding and the role of the funders for the present study and, if applicable, for the original study on which the present article is based | 13 | The authors are thankful to the Noguchi Memorial Institute for Medical Research (NMIMR) for funding the study through NMIMR Office for Research Support Fund (Fund ID- EC/P25421/03) to JA. The funders had no role in the study design, data collection and analysis, decision to publish, or preparation of the manuscript. |

*Give information separately for cases and controls in case-control studies and, if applicable, for exposed and unexposed groups in cohort and cross-sectional studies.

**Note:** An Explanation and Elaboration article discusses each checklist item and gives methodological background and published examples of transparent reporting. The STROBE checklist is best used in conjunction with this article (freely available on the Web sites of PLoS Medicine at http://www.plosmedicine.org/, Annals of Internal Medicine at http://www.annals.org/, and Epidemiology at http://www.epidem.com/). Information on the STROBE Initiative is available at www.strobe-statement.org.
