# Supplementary material for: Assessing the performance of regular surgical nose masks as a sampling method for SARS-CoV-2 detection in a cross-sectional study
Source: PLoS One. 2023 Oct 17;18(10):e0293001. doi: 10.1371/journal.pone.0293001 (PMC10581487; doi:10.1371/journal.pone.0293001)
Supplement: S2 Table — (DOCX) [file pone.0293001.s003.docx]

**Supplementary Information**

**Assessing the performance of regular surgical nose masks as a sampling method for SARS-COV-2 detection in a cross-sectional study**

Millicent Opoku et al.

*Corresponding author: [jakorli@noguchi.ug.edu.gh](mailto:jakorli@noguchi.ug.edu.gh)

**S2 Table: Results for linear regression model for Ct values between test methods**

Call:

lm(formula = **retro_ct ~ swab_ct,** data = res)

Residuals:

Min 1Q Median 3Q Max

-13.1430 0.0072 0.4460 1.3208 2.5293

Coefficients:

Estimate Std. Error t value Pr(>|t|)

(Intercept) 41.21714 0.82374 50.036 < 2e-16 ***

swab_ct 0.08390 0.02309 3.634 0.000441 ***

---

Signif. codes: 0 ‘***’ 0.001 ‘**’ 0.01 ‘*’ 0.05 ‘.’ 0.1 ‘ ’ 1

Residual standard error: 2.314 on 101 degrees of freedom

Multiple R-squared: 0.1156, Adjusted R-squared: 0.1069

F-statistic: 13.2 on 1 and 101 DF, p-value: 0.0004414

Call:

lm(formula = **new_ct ~ swab_ct**, data = res)

Residuals:

Min 1Q Median 3Q Max

-25.0067 0.4833 0.5752 0.7082 0.8803

Coefficients:

Estimate Std. Error t value Pr(>|t|)

(Intercept) 43.93115 1.03828 42.311 <2e-16 ***

swab_ct 0.01301 0.02910 0.447 0.656

---

Signif. codes: 0 ‘***’ 0.001 ‘**’ 0.01 ‘*’ 0.05 ‘.’ 0.1 ‘ ’ 1

Residual standard error: 2.916 on 101 degrees of freedom

Multiple R-squared: 0.001975, Adjusted R-squared: -0.007907

F-statistic: 0.1999 on 1 and 101 DF, p-value: 0.6558
