# Supplementary material for: Assessing the performance of regular surgical nose masks as a sampling method for SARS-CoV-2 detection in a cross-sectional study
Source: PLoS One. 2023 Oct 17;18(10):e0293001. doi: 10.1371/journal.pone.0293001 (PMC10581487; doi:10.1371/journal.pone.0293001)
Supplement: S3 Table — (DOCX) [file pone.0293001.s004.docx]

**Assessing the performance of regular surgical nose masks as a sampling method for SARS-COV-2 detection in a cross-sectional study**

Millicent Opoku et al.

*Corresponding author: [jakorli@noguchi.ug.edu.gh](mailto:jakorli@noguchi.ug.edu.gh)

**S3 Table: Detailed statistical estimates for sensitivity, specificity and predictive prevalence analyses of nose masks compared to naso-oropharyngeal swabs.** ‘Test’ refers to the NOP swabs and ‘outcome’ refers to masks. Analyses were performed with package *epiR* in R-software.

|  | **Retro masks** | | | **New masks** | | |
| --- | --- | --- | --- | --- | --- | --- |
| **statistic** | **est** | **lower** | **upper** | **est** | **lower** | **upper** |
| ap | 0.6407767 | 0.54027182 | 0.7329574 | 0.6407767 | 0.54027182 | 0.7329574 |
| tp | 0.09708738 | 0.04754642 | 0.1713063 | 0.04854369 | 0.01594759 | 0.1096561 |
| se | 0.9 | 0.55498388 | 0.9974714 | 0.8 | 0.28358206 | 0.9949492 |
| sp | 0.38709677 | 0.28783093 | 0.4937614 | 0.36734694 | 0.27219429 | 0.4707477 |
| diag.ac | 0.4368932 | 0.3393545 | 0.5381561 | 0.38834951 | 0.2938723 | 0.4894225 |
| diag.or | 5.68421053 | 0.6907307 | 46.7769123 | 2.32258065 | 0.24987847 | 21.5880178 |
| nndx | 3.48314607 | -6.3619225 | 2.0356945 | 5.97560976 | -2.2511183 | 2.1473195 |
| youden | 0.28709677 | -0.1571852 | 0.4912328 | 0.16734694 | -0.4442236 | 0.4656969 |
| pv.pos | 0.13636364 | 0.06429819 | 0.2431413 | 0.06060606 | 0.01675822 | 0.1479676 |
| pv.neg | 0.97297297 | 0.8583969 | 0.999316 | 0.97297297 | 0.8583969 | 0.999316 |
| lr.pos | 1.46842105 | 1.12969274 | 1.908714 | 1.26451613 | 0.79547831 | 2.0101127 |
| lr.neg | 0.25833333 | 0.03954192 | 1.6877309 | 0.54444444 | 0.0925335 | 3.2033777 |
| p.rout | 0.3592233 | 0.26704257 | 0.4597282 | 0.3592233 | 0.26704257 | 0.4597282 |
| p.rin | 0.6407767 | 0.54027182 | 0.7329574 | 0.6407767 | 0.54027182 | 0.7329574 |
| p.tpdn | 0.61290323 | 0.50623859 | 0.7121691 | 0.63265306 | 0.52925235 | 0.7278057 |
| p.tndp | 0.1 | 0.00252858 | 0.4450161 | 0.2 | 0.00505076 | 0.7164179 |
| p.dntp | 0.86363636 | 0.75685867 | 0.9357018 | 0.93939394 | 0.85203244 | 0.9832418 |
| p.dptn | 0.02702703 | 0.00068403 | 0.1416031 | 0.02702703 | 0.00068403 | 0.1416031 |

| tp | True prevalence. |
| --- | --- |
| ap | Apparent prevalence. |
| se | Diagnostic test sensitivity. |
| sp | Diagnostic test specificity. |
| diag.ac | Diagnostic accuracy (the correctly classified proportion). |
| diag.or | Diagnostic odds ratio. |
| nndx | The number needed to diagnose. |
| youden | Youden's index. |
| pv.pos | Positive predictive value. |
| pv.neg | Negative predictive value. |
| lr.pos | Likelihood ratio of a positive test. |
| lr.neg | Likelihood ratio of a negative test. |
| p.rout | The proportion of subjects with the outcome ruled out. |
| p.rin | The proportion of subjects with the outcome ruled in. |
| p.tpdn | The proportion of true outcome negative subjects that test positive (false T+ proportion for D-). |
| p.tndp | The proportion of true outcome positive subjects that test negative (false T- proportion for D+). |
| p.dntp | The proportion of test positive subjects that are outcome negative (false T+ proportion for T+). |
| p.dptn | The proportion of test negative subjects that are outcome positive (false T- proportion for T-). |
